# Gene-Level Shift in Response to Synthetic Nitrogen Addition Promotes *Larix olgensis* (Ussurian Larch) Growth in a Short-Term Field Trial

**DOI:** 10.3390/life15091403

**Published:** 2025-09-04

**Authors:** Muhammad Jamal Ameer, Yushan Liu, Siyu Yan, Tongbao Qu

**Affiliations:** College of Forestry and Grassland, Jilin Agricultural University, Changchun 130118, China; jameer6524@gmail.com (M.J.A.); 18943478100@163.com (Y.L.); 16643179269@163.com (S.Y.)

**Keywords:** *Larix olgensis*, ammonium nitrogen, nitrate nitrogen, gene abundances, qPCR

## Abstract

Climate change and injudicious nitrogen addition alter the soil physico-chemical properties and microbial activity in oligotrophic forest soil, which disrupts the nitrogen cycle balance. Nevertheless, recommended fertilizer forms and levels are considered to be crucial for stable nitrogen application. We established a short-term field trial for the first time using a randomized complete block design under the yellow larch forest, with six treatments applied, including urea CO(NH_2_)_2_, ammonium chloride NH_4_Cl, and sodium nitrate NaNO_3_ at concentrations of 10 and 20 kg N hm^−2^ yr^−1^, each extended by three replicates. The gene abundances were measured using quantitative PCR (qPCR), in which the abundance levels of AOA (*amoA*) and *nirS* were higher under high CO(NH_2_)_2_ 2.87 × 10^10^ copies g^−1^ dry soil and low NO_3_^−^ 8.82 × 10^9^ copies g^−1^ dry soil, compared to CK, representing 2.8-fold and 1.5-fold increases, respectively. We found niche partitioning as revealed despite AOA (*amoA*) increasing in number, AOB (*amoA*) contributing more to ammonia oxidation while *nirS* proved opportunistic under stress conditions. This was supported by distinct significant correlations among factors, in which soil urease enzymatic activity (S-UE) was associated with AOA (*amoA*) and *nirK*, while AOB (*amoA*) and *nirS* positively correlated with NH_4_^+^ content and soil potential of hydrogen (pH), respectively. Among the applied treatments, high-level NO_3_^−^ increased total nitrogen content and had a significant effect on soil N-acetyl-β-d-glucosaminidase (S-NAG) and soil acid protease (S-ACPT) activity. In summary, we observed an increase in *Larix olgensis* growth with high nitrogen retention.

## 1. Introduction

Nitrogen plays a vital role in supporting plant growth and ecosystem function [1]. The complexity of nitrogen transformation in different environments is still under debate, and little information is available for oligotrophic forest soil [2]. In past studies, the differential effect of applied synthetic nitrogen on nitrogen cycling gene abundances was discussed, with results of nitrogen availability or loss [3,4]. Globally, nitrogen addition has contributed to N_2_O emissions by 30% per year over the past four decades [5]. A meta-analysis revealed that unbalanced nitrogen fertilizer addition promotes N_2_O emission, stimulating partial denitrification [6]. Ammonia oxidizers and denitrifiers are key drivers for nitrification and denitrification [2,7]. The nitrification process comprises two steps; one is ammonia oxidation, which involves ammonia-oxidizing archaea (AOA) and ammonia-oxidizing bacteria (AOB) [8], and the second is nitrite oxidation, which involves nitrite-oxidizing bacteria (NOB) [9].

Previously, Ouyang et al. focused their nitrogen application study on nitrification gene abundances in agricultural soil [10]. Similarly, Liu et al. also observed the synthetic nitrogen effect on nitrifying gene abundances in cropping soil [11], with gaps left for oligotrophic forest soil nitrogen cycling. Few studies have explored ammonia oxidation in terrestrial forest soil. For example, a study by Prosser et al. described AOA exceeding AOB in abundance upon high N addition [12], and it was also observed that AOB dominated in abundance over AOA [13]. Similarly, according to Stopnisek et al., inorganic nitrogen increased AOB abundances, while AOA abundance was increased by organic nitrogen addition in oligotrophic forest soil [14]. However, which N fertilizer form is favored between the two ammonia oxidizers at the optimum level has still not been elaborated clearly. However, it is evident from past studies that the source of applied chemical nitrogen and its level contributed to AOA and AOB distribution, as high NH_4_^+^ stimulated AOB abundances [15]. Likewise, low NH_4_^+^ increases AOA abundances [16]. In a microcosm study, Rutting et al. demonstrated that low NH_4_^+^ increases AOA abundances while high NH_4_^+^ increases AOB abundances [8]. In the same way, high CO(NH_2_)_2_ application increases AOB abundances while low level CO(NH_2_)_2_ addition increases AOA abundances [17]. These studies elucidated the different effects of applied nitrogen fertilizer levels on ammonia oxidizer abundances.

In addition to the level of N addition, the substrate form of applied nitrogen also induces different effects. For instance, a study reported that the NH_3_^+^ form is a crucial factor as a substrate between AOA and AOB [18], differentiating in abundances. Similar to this, Sterngren et al. also elaborated on the difference in nitrogen substrate form between the two ammonia oxidizers [19]. In addition to the direct effects of substrate nitrogen form and level on AOA and AOB, ammonia oxidizers are also indirectly influenced by changes in soil pH, which is a key factor for understanding nitrification in oligotrophic forest soils [20]. Different past studies have described how AOA dominates in acidic soil ammonia oxidation, but synthetic nitrogen addition changes the conditions, shifting toward AOB dominance in ammonia oxidation [20,21]. Some studies also revealed different effects, such as AOB dominance in low-pH soil [22] and AOA dominance in higher-pH soil [21]; such discrepancies need to be settled. Therefore, it is critical to understand the applied N effect with different forms and concentrations on ammonia oxidizer abundances, for sustainable N transformation in *L. olgensis* soil.

Denitrification is a reduction process in which a denitrifier reduces nitrogen in one form into another under anaerobic conditions, utilizing NO_3_^−^ as a substitute for atmospheric O_2_ [23], causing NO_3_^−^ reduction to NO_2_, NO, N_2_O, and N_2_ driven by functional genes *narG*/*napA*, *nirK*/*nirS*, *norB,* and *nosZ,* respectively [23]. Ameer et al. described how differences in gene abundances occur due to variations in nitrogen forms and levels of substrate added [2]. For example, studies explained that high CO(NH_2_)_2_ and high NO_3_^−^ increase *nirK* gene abundances while *nirS* gene abundances decrease [24,25]. Similarly, according to Veraart et al., high NO_3_^−^ increases *nirK* gene abundances and low NO_3_^−^ increases *nirS* abundances [26]. *nosZ* gene abundances decrease in both low- and high-level CO(NH_2_)_2_ [27] while increasing in high NO_3_^−^ conditions [28]. A previous study also described how the main gradient factor regarding denitrifier abundances is soil pH, and these genes responded differently to the applied N sources due to changes in soil pH [29]. While in a past study it was also reported that carbon sources stimulate denitrifier abundances as a substrate [30], whereas a study recently demonstrated that NO_3_^−^ concentration is a dominant factor between denitrifier and denitrification [31]. Hence, it is crucial to understand the direct or indirect applied nitrogen effects on denitrifier gene abundances for the identification of eco-friendly nitrogen transformation pathways. Hallin et al. explained that it is a strong possibility that gene abundances of functional groups are related to potential rates of nitrogen conversion processes [32]. Peterson et al. also described how abundances of functional genes are very important variables to indicate the potential rate of N conversion [13]. Therefore, N application in oligotrophic forest soil based on knowledge of gene pathways will lead to enhanced sustainable forest production [33].

AOA, AOB, and *nirK*, *nirS* and *nosZ* were selected to analysis absolute gene abundances as the ammonia oxidizers and denitrifier, respectively. To identify the correlation of gene abundances with soil’s physico-chemical properties and enzyme activity, we determined soil properties and enzyme assay analysis, and additionally, to discover the possible redundant role of genes in oligotrophic forest soil, we observed pathway trends between quantitative qPCR abundances and shot-gun metagenomics KEGG pathway relative abundances. The findings of this study allow us to understand possible changes in the gene pathway in response to applied nitrogen, which will lead to the right choice for chemical nitrogen fertilizer application. The questions to be answered in this study are as follows: 1 Do different nitrogen forms and levels impact nitrification and denitrification processes, thereby increasing the abundance of key genes? 2 Do different forms of nitrogen addition have different effects? 3 Is the effect related to the level of nitrogen added?

## 2. Materials and Methods

### 2.1. Experimental Site and Design

Research was conducted in *L. olgensis* tree soil, located on a hill situated on the bank of the lake at Jilin Agricultural University (43°05′–45°15′ N; 124°18′–127°05′ E), Changchun, Jilin Province, P. R China. The site has a monsoon season with medium latitude, and the annual temperature is 4.8 °C. Soil pH is 5.6 and mean annual precipitation is 570.3 mm. High-precipitation months are from July to August. Major understory species include *L. olgensis* (Ussurian larch), *B. inermis* (awnless brome), *C. majus* (greater celandine) and *V. prionantha* (Japanese Violet).

This study is based on a one-time field fertilization experiment conducted over a (short-term) period of 1 year (June to August 2024). The nitrogen addition and interruption test before this trial proceeded as follows: The experiment from 2018 to 2021 comprised three N addition treatments, including control (CK: 0 kg N hm^−2^ yr^−1^), low nitrogen (LN: 10 kg N hm^−2^ yr^−1^) and high nitrogen (HN: 20 kg N hm^−2^ yr^−1^), with NaNO_3_^−^ as the nitrogen fertilizer applied. In August 2021, soil was sampled after 4-year nitrogen fertilizer treatment and was not treated again to maintain soil natural recovery until 2023, meaning 2 years of N interruption.

**Figure 1:**
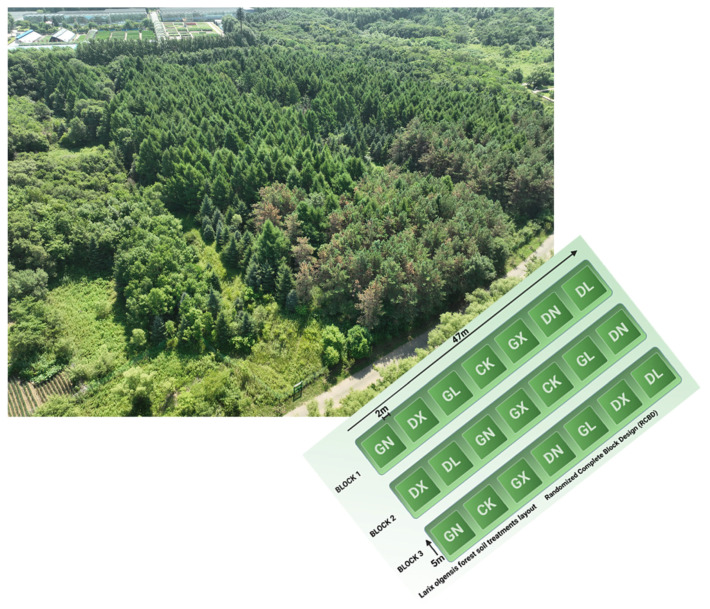
Schematic presentation of randomized complete block design field trial and plots of *Larix olgensis* forest soil with aerial view. GN: High-level urea treatment. DN: Low-level urea treatment. GL: High-level ammonium chloride treatment. DL: Low-level ammonium chloride treatment. GX: High-level sodium nitrate treatment. DX: Low-level sodium nitrate treatment. CK: Control treatment.

After taking a sample of the 2-year N interruption in July 2023, the site was treated in June 2024 with chemical N addition, which included two N levels, low (10 kg N hm^−2^ yr^−1^) and high (20 kg N hm^−2^ yr^−1^), and three forms of N addition as treatments: CO(NH_2_)_2_, NH_4_Cl and NaNO_3_. Different low and high levels of applied nitrogen were chosen according to the history of this experimented soil, and various forms were selected for the comparative analysis of nitrogen effect on gene abundances and soil properties. Each N treatment was repeated 3 times (3 × 2 × 3), for a total of 18 sample plots. Each plot measured 5 × 5 m and was established in RCBD (Randomized Complete Block Design), as shown in Figure 1. Three plots were selected as the control (CK).

Samples were taken in August 2024 from each nitrogen form treatment plots, from five different places, and were divided into two parts: one for analysis of soil properties and the other for soil microbial gene quantification and enzymatic activities. Soil samples were sieved (<2 mm) and kept at −20 °C for standard analysis.

### 2.2. Chemical Test Methods of Soil Properties

Soil pH: Soil pH was measured as in the following methodology [34]. The air-dried soil was passed through a 2 mm sieve and 10 g of soil sample was weighed and then placed in a 50 mL beaker. After this, 25 mL deionized water was added (1:2.5 soil to water ratio) and the suspension was stirred for 20 min and left to stand for 30 min. After, the magnetic pH meter was placed on the bulb of the upper glass electrode immersed in the supernatant of the soil sample to measure. When the reading was stable, the soil pH value was recorded.

Water content: First, 10 g of soil sample was taken into an aluminum box, then the wet weight of the soil sample was determined, it was dried at 105 °C for 24 h, and then the dry weight was measured to calculate the moisture content.

Electrical Conductivity: EC is measured by an EC meter. We weighed 10 g of soil and added 50 mL deionized water for accuracy (1:5 ratio), and after this stirred and let it stand for 30 min. Then, the extract was filtered and an EC probe inserted for reading.

NH_4_^+^ concentration: A spectrophotometer was used to measure the concentration. We extracted NH_4_^+^ using 2 M KCl, at a soil/solution ratio of 1:5, shaken it for 30 min and then centrifuged at 4000 rpm for 10 min. The supernatant was filtered and NH_4_^+^ concentration was determined colorimetrically using the indophenols blue method.

NO_3_^−^ concentration: Soil sample was extracted using 2 M KCl reagent, with a soil/solution ratio of 1:5, shaken for 30 min and then centrifuged at 4000 rpm for 10 min to separate the supernatant.

Total Nitrogen: TN content measured using the semi-Kjeldahl method [35].

Total Carbon: Organic content of carbon was measured with the H_2_SO_4_-K_2_Cr_2_O_7_ wet oxidation method [36].

### 2.3. Quantification of Functional Gene Abundances Using Quantitative PCR

To calculate functional gene abundances, quantitative PCR was used. AOA, AOB including *nirK*, *nirS* and *nosZ* genes, with F and R primers were used for quantification. For quantitative measure of archaeal and bacterial *amoA* gene, the primers were as follows: Arch-*amoA*F (5′-**S**TAATGGTCTGGCTTAGACG-3′) and Arch-*amoA*R (5′-GCGGCCATCCATCTGTATGT-3′) [37]; AmoA1F (5′-GGGG**H**TT**Y**TACTGGTGGT-3′) and AmoA2R (5′-CCCCTC**K**G**S**AAAGCCTTCTTC-3′) [38]. For *nirS*, they were Cd3aF (5′-GT**S**AACGT**S**AAGGA**R**AC**S**GG-3′) and R3cd (5′-GA**S**TTCGG**R**TG**S**GTCTTGA-3′) [39]; nirK1F (5′-GG**M**ATGGT**K**CC**S**TGGCA-3′) and nirK5R (5′-GCCTCGATCAG**R**TT**R**TGG-3′) [40]; and nosZFb (5′-AACGCCTA**Y**AC**S**AC**S**CTGTTC-3′) and nosZRb (5′-TCCATGTGCAG**N**GC**R**TGGCAGAA-3′) [39]. Bold letters denote degenerate positions: M = A/C, R = A/G, S = G/C, Y = C/T, K = G/T, B = G/C/T, H = A/C/T, and N = A/G/C/T. For qPCR, a stock solution of plasmid in the range of 10^4^ to 10^8^ copies was created for the standard curve of *nirK* and AOA *amoA*. Similarly, for *nirS* and AOB *amoA*, 10^5^ to 10^9^ copies were created. Microbial DNA extraction was performed using the Ultra-Pure Soil Genomic DNA Rapid Extraction Kit (Adelai Biotechnology Co., Ltd., Beijing, China). DNA was quantified using a Nano Drop TM ND-1000 UV-Vis Spectrophotometer (Thermo-Scientific, Rockwood, TN, USA). The qPCR reaction was carried out using the LightCycler^®^ 480 thermocycler, Mannheim, Germany. The qPCR reaction system comprised 10 μL SYBR^®^PremixEx Taq (Takara, Dalian, China). For sample DNA, 1 μL soil, forward primer 1 μL 10 μmol/L, reverse primer 1 μL 10 μmol/L, and 7.0 μL H_2_O were used. Conditions for reaction were 95 °C denaturation at 2 min followed by 95 °C for 5 s, and annealing for 15 s at 60 °C, 45 cycles, maintained at 72 °C for 20 s.

### 2.4. Enzyme Assay

Activities of five enzymes involved in nitrogen cycling were measured. Soil enzyme activities were determined using colorimetric assays. Urease activity was measured using the phenol-sodium hypochlorite colorimetric method [41]; acid protease activity (ACPT) was measured with the colorimetric method, using acetate buffer pH = 5.0 [41]. N-acetyl-β-d-glucosaminidase (NAG) activity was determined by using a colorimetric method based on p-nitrophenyl-N-acetyl-β-d-glucosaminidase (pNP-NAG) as the substrate. Briefly, 1 g of fresh soil was mixed with 4 mL of sodium acetate buffer (pH 5.5) and vortexed for 30 s. Then, 1 mL of 2 mM pNP-NAG substrate solution was added to the mixture and incubated at 37 °C for 1 h. The reaction was stopped by adding 1 mL of 0.5 M NaOH, followed by centrifugation at 10,000 rpm for 5 min. Absorbance of the supernatant was measured at 405 nm using a spectrophotometer, and enzyme activity was calculated using a standard curve of p-nitrophenol (pNP). For nitrite reductase and nitrate reductase, quantitative analysis was followed by benzene sulfonic acid–acetic acid–a-naphthylamine with the colorimetric method. Soil nitrate reductase (S-NR) was calculated by measuring the absorption peak at 540 nm, and soil nitrite reductase (S-NiR) catalyzed the conversion of nitrite (NO_2_^−^) into nitric oxide (NO), thereby decreasing the concentration of nitrite in the sample. Residual nitrite was first subjected to diazotization with p-aminobenzenesulfonic acid, forming a diazonium compound, which was subsequently coupled with an α-amine to produce a reddish-purple azo dye. The intensity of the resulting color was quantified by measuring absorbance at 540 nm [42].

### 2.5. Statistical Analyses

Statistical analysis was conducted using R (Foundation for Statistical Computing). Similarly, data analysis was carried out with SPSS 26.0 and a graph was made using GraphPad Prism 9 with origin 2021 software. Non-normal data was normalized for a one-way analysis of variance (ANOVA) (for which *p*-value (*p* < 0.05) was considered significant as compared to the control group) using a Shapiro–Wilk test, and homogeneity of variance was determined with Levene’s test. Tukey’s post hoc test was used (*p* < 0.05) for pairwise multiple comparison and controlling error rate. Fertilizer treatment effects on soil physico-chemical properties and nitrification/denitrification gene abundances were analyzed by mean and standard deviation (SD), significant difference, F value, and *p*-value separately for each variable to analysis whether there were significant differences among the treatments groups. Correlation analysis among soil properties, enzymes activities and gene abundance was performed by Pearson correlation analysis. Canoco 5.0 redundancy (RDA) analysis was applied to discover the most-affected factor influences.

## 3. Results

### 3.1. Effect of Nitrogen Addition on Soil Physico-Chemical Properties

For the applied form and level of nitrogen, each treatment had significantly positive/negative correlations with soil properties.

As described in Table 1, all treatments significantly reduced soil pH, except low and high NO_3_^−^ (DX and GX), which increased it by 6.7% and 4.1%, respectively, while DX showed a strong significant correlation with soil pH (*p* < 0.01). In contrast to our study result, a past study in *L. olgensis* soil (2023) showed a decreasing trend of soil pH with lower and higher NO_3_^−^ application, which reflected the stability of the soil and a decrease in soil acidification in this *L. olgensis* trial. Similarly, as compared to the control group and low NO_3_^−^ (DX), all treatments increased total carbon (TC), while past soil fertilization and N interruption experiments in *L. olgensis* soil (2023) showed a decrease in total carbon content. Ammonium content was significantly higher under the GL treatment and increased at 1.54 mg kg^−1^, while nitrate concentration increased under GX, with high NO_3_^−^ treatment, to 16.38 mg kg^−1^. Total nitrogen (TN) increased under lower and higher NO_3_^−^ application, by 54.8% and 80.6%, respectively. The GL treatment with high NH_4_^+^ was statistically significant with electrical conductivity (EC) (*p* < 0.01) and soil water content (SWC) was only positively correlated with DX treatment (*p* < 0.01), and all other treatments were not statistically significant for soil water content (SWC). Significant interaction effects indicate optimized nitrogen application, enhanced nutrient content and soil stability.

### 3.2. Effect of Nitrogen Addition on Ammonia Oxidizer and Denitrifier Abundances

**Figure 2:**
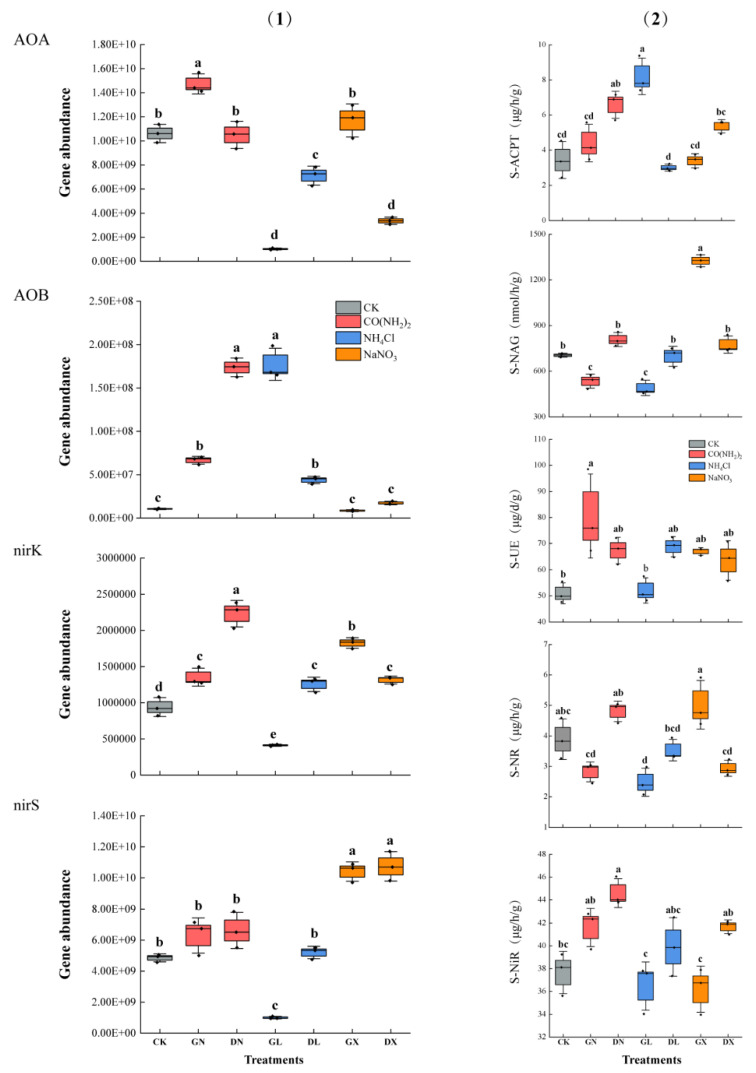
Subfigure (**1**) comprised detail of ammonia oxidiserz and denitrifiers abundance quantitatively measured by (qPCR) while subfigure (**2**) highlights the impact of different treatments used on soil enzymatic activities. Each different-colored box represents types of specific treatments applied, while horizontal line letters indicate treatments: CK as control, GN as high CO(NH_2_)_2_, DN as low CO(NH_2_)_2_, GL as high NH_4_^+^, DL as low NH_4_^+^, GX as high NO_3_^−^ and DX as low NO_3_^−^, respectively. Similarly, S-NiR: soil nitrite reductase; S-NR: soil nitrate reductase; S-UE: soil urease; S-NAG: soil N-acetyl-β-d-glucosaminidase; S-ACPT: soil acid protease (as abbreviated). Among treatments, the same letter denotes non-significant effect.

Different forms and levels of applied nitrogen had significant effects on functional genes. Figure 2(1) shows marked treatments with notable differences. Bacterial *amoA* shows significantly higher abundances upon high NH_4_^+^ application under GL treatment (*p* < 0.01), and increased from 1.63 × 10^7^ to 3.59 × 10^8^ copies g^−1^ dry soil. Meanwhile, archaeal *amoA* only significantly increased at high CO(NH_2_)_2_ GN treatment as compared to CK (*p* < 0.05). Among the denitrifier abundances, the *nirS* gene increases at low NO_3_^−^ treatment, and did so statistically significant with DX treatment (*p* < 0.01). Meanwhile, the *nirK* gene was only significantly correlated with low CO(NH_2_)_2_ DN treatment and increased from 1.28 × 10^6^ copies g^−1^ dry soil to 3.83 × 10^6^ copies g^−1^ dry soil. In *L. olgensis* forest soil, we could not find the *nosZ* gene through qPCR analysis, which may be due to the predominance of *nirK* genes in these soils’ metabolic pathways, which can function independently without the requirement of supporting genes as compared to *nirS.*

### 3.3. Effect of Nitrogen Addition on Soil Enzymatic Activities

Nitrogen addition has positive and negative effects on soil enzymes’ activities according to the form and level of N added. The interaction between N forms and enzyme activity of S-UE, S-ACPT and S-NR was significant. Compared to control, high-level NO_3_^−^ has a significant effect on S-NR and S-NAG activity (*p* < 0.05), while high NH_4_^+^ only significantly increased S-ACPT activity (*p* < 0.01) and high CO(NH_2_)_2_ stimulated S-UE activity. The promotion of enzymatic activities was only significantly higher correlatively with the high level of applied nitrogen form. Neither NH_4_^+^ nor NO_3_^−^ had a significant effect on S-UE activity Figure 2(2).

### 3.4. Relationship Between N Addition Effect on Gene Abundances, Soil Physico-Chemical Properties and Enzymes Activities

**Figure 3:**
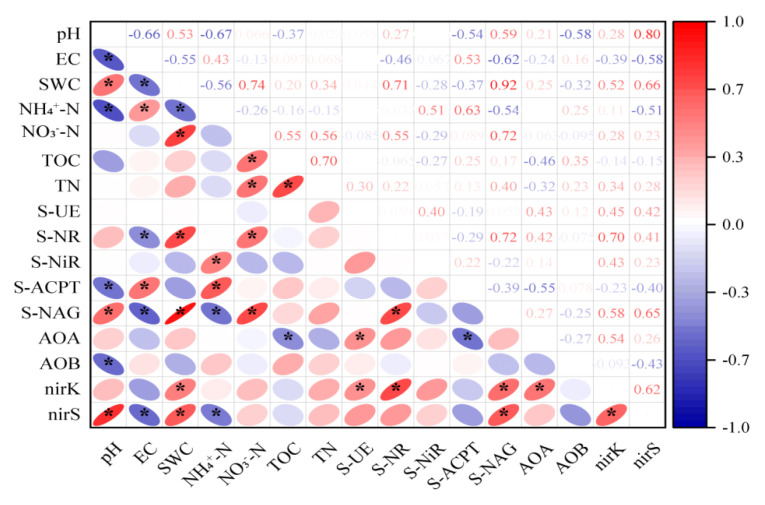
Correlation heat map of soil properties, enzymes activities and microbial gene abundances, shown in red for positive correlation and blue for negative correlation; * indicates *p* ≤ 0.05. S-NiR: soil nitrite reductase, EC: electrical conductivity, SWC: soil water content, TC: total carbon, TN: total nitrogen, S-NR: soil nitrate reductase, S-UE: soil urease, S-NAG: soil N-acetyl-β-d-glucosaminidase, S-ACPT: soil acid protease, AOA: ammonia oxidizing archaea, AOB: ammonia oxidizing bacteria (as abbreviated).

**Figure 4:**
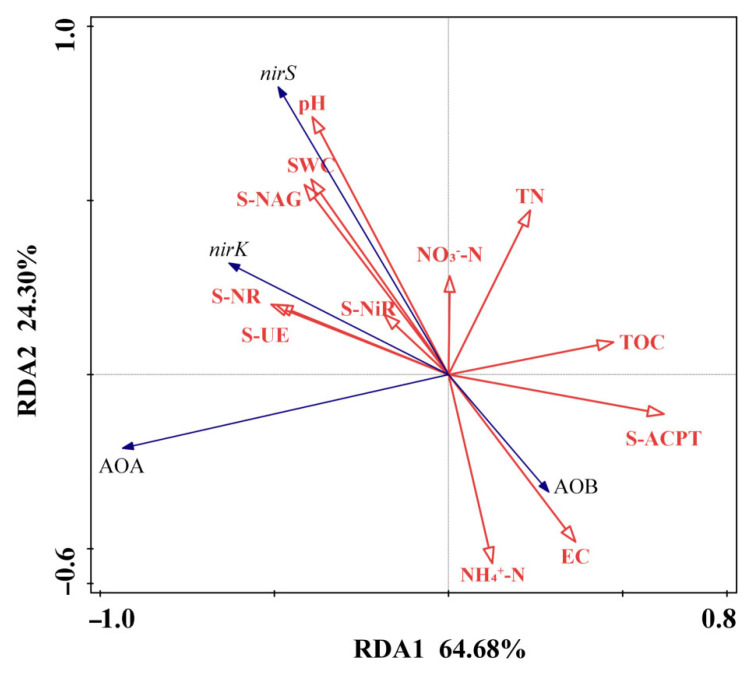
Redundancy analysis diagram of the abundance of functional genes and soil environmental factors. EC: electrical conductivity, SWC: soil water content, TOC: total organic carbon, TN: total nitrogen, S-NR: soil nitrate reductase, S-UE: soil urease, S-NAG: soil N-acetyl-β-d-glucosaminidase, S-ACPT: soil acid protease, AOA: ammonia oxidizing archaea, AOB: ammonia oxidizing bacteria (as abbreviated).

The correlation analysis revealed that soil pH showed a strong positive correlation with *nirS* (*p* ≤ 0.01), suggesting that pH plays a critical role in shaping denitrifying communities. Similarly, NO_3_^−^ had statistically significant results with total nitrogen (*p* ≤ 0.05) and was positively correlated with *nirS*, as shown in Figure 3, which indicates complete denitrification. The analysis of most influencing factors for enzyme activities, soil properties and gene abundance is explained in Figure 4.

As can be seen from Figure 4, the contribution rates of axis 1 and axis 2 are 64.68% and 24.30%, respectively. The acute angles between the arrow of the *nirS* and the arrows of soil environmental factors such as pH, SWC, and S-NAG indicate that the abundance of *nirS* is positively correlated with these soil environmental factors. The obtuse angle between the arrow of the AOA and the arrows of soil environmental factors such as TN and the acute angles between the AOA arrow and S-UE, S-NAG, and SWC suggest that the abundance of AOA is negatively correlated with factors such as TN and positively correlated with soil factors such as S-UE. The smallest angle between the *nirK* and S-UE indicates that S-UE has the greatest impact on *nirK*. The abundance of AOB is positively correlated with soil factors such as NH_4_^+^-N, EC and S-ACPT, among which EC has the most significant impact.

**Figure 5:**
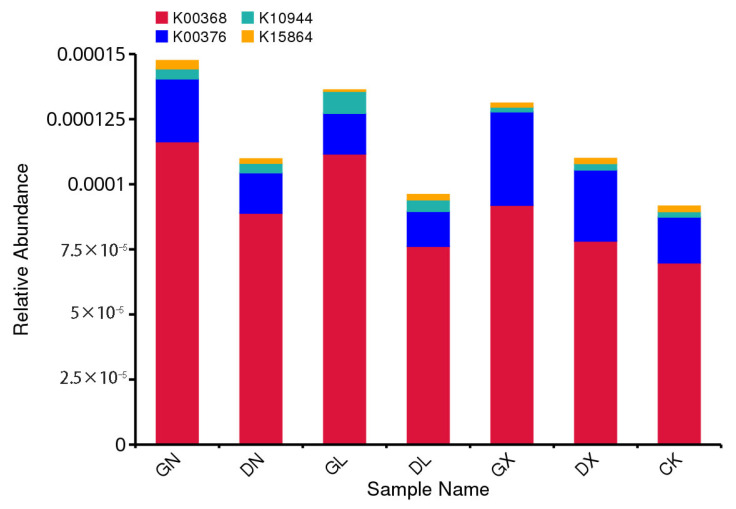
Abundance map of KEGG pathway. Note: Vertical lines represent proportional values of KEGG pathway relative abundances, red color bars and K0 number reflect nir gene *nirK*, blue color is *nosZ* gene, green color is *amoA* gene, and yellow is *nir* gene *nirS*, in order. Horizontal letters represent names of treatments: GN is high CO(NH_2_)_2_, DN is low CO(NH_2_)_2_, GL is high NH_4_^+^, DL is low NH_4_^+^, GX is high NO_3_^−^, and DX is low NO_3_^−^, respectively.

Forest soil nitrogen cycle microbial pathways are diverse and complex due to the involvement of different unknown microbial communities. Denitrifying genes *nirK* and *nirS* are among them. However, previous studies have shown that the *nirS* gene has a robust metabolic system and can conserve energy under stress conditions, while the *nirK* gene is independent in its role [43]. In order to understand denitrifying gene pathways or redundant roles under mineral nitrogen addition in *L. olgensis* forest soil, we analyzed shot-gun metagenome relative abundances. Relative abundances of genes’ metabolic pathways are shown in Figure 5.

As per the analysis, the *nirK* gene seemed to adapt to a more organic environment relative to *nirS* and was less efficient in adapting to the stress condition. Our results, obtained through dual methodology advance information on the niche partitioning of denitrifying communities, in response to environmental changes under oligotrophic soil.

## 4. Discussion

This study was conducted as a short-term field experiment to investigate the early responses of microbial gene abundances to the different N forms and levels in *L. olgensis* forest soil. While the dynamic nature of forest soil suggests multiple times long-term field experiments at gene expression level, which may provide clearer insight on the N transformation process. As the effect of different synthetic N forms and levels on the key gene abundances of the nitrogen cycle were analyzed for the first time at field level so, the current results offer insights into the initial shifts using targeted genetic analysis and metagenomic analysis in N cycling processes. The findings can serve as a foundation for future long-term studies employing more quantitative and comprehensive techniques such as RT-qPCR and meta-transcriptomics.

### 4.1. Effect of Nitrogen Addition on Soil Properties and Enzymatic Activities

Synthetic nitrogen fertilizers provide readily available inorganic nitrogen forms (NH_4_^+^/NO_3_^−^), enabling rapid uptake by plants and microorganisms compared with slower mineralization. Oligotrophic forest soil in terrestrial ecosystems is highly sensitive to different forms and levels of nitrogen application. N additions increased total nitrogen through the addition of NH_4_^+^ and NO_3_^−^ fertilizers, or caused losses, changing soil microbial activities. Our study revealed that varying nitrogen forms and levels significantly altered soil functional properties and contributed to soil nutrient richness. DX (low NO_3_^−^) significantly increased soil water content, which indicate less NO_3_^−^ leaching, while a study by Qu et al. observed a decrease in water content under lower NO_3_^−^ addition [44]. In the study by Qu et al., the NO_3_^−^ content increased and soil water content decreased under low NO_3_^−^ addition, while this study’s results were different, with increasing soil water content indicating higher NO_3_^−^ uptake and complete denitrification causing sustainable NO_3_^−^ consumption. Similarly, (GL) and (GX) treatments in our study increased organic carbon content, which correlates with a study by Fang et al. [45] in which applied ammonium nitrogen and nitrate nitrogen had significant effects on total carbon; however, in a previous *L. olgensis* soil trial by Qu et al., low and high NO_3_^−^ addition decreased total carbon [44]. This contradiction in results is possibly due to higher NO_3_^−^ accumulation and increase in soil pH in our study, while the study by Qu et al. showed a decrease in soil pH. The rationale is that soil pH is a key factor for microbial activity, which increases upon increasing soil pH. Although usually nitrogen addition causes soil acidification and decreases soil pH, as described by past studies [44,46]. However, this difference in our study was due to two causes: 1. This soil before fertilizer treatment passed through 2 years of nitrogen interruption, which alleviated soil acidification [47]. 2. As compared to the study by Qu et al., our study stabilized soil pH by promoting NO_3_^−^ assimilation [44]. Overall, GX (higher NO_3_^−^) significantly increased NH_4_^+^, NO_3_^−^ and total nitrogen (TN) content (*p* ≤0.05), which was also increased in a study by Lu et al. [48]. This nutrient improvement caused by applied nitrogen levels beneficial impact on microbial enzymatic activities by promoting N-acetyl-glucosaminidase (S-NAG) activity, (S-ACPT) activity and total NH_4_^+^ content through the addition of higher-NO_3_^−^ fertilizer concentration. Yan et al. observed a similar trend in their results, in which (S-NAG) activity significantly increased with higher-NO_3_^−^ fertilizer. Similarly, Qu et al. found an increasing impact on (S-NAG) activity and (S-ACPT) activity under high NO_3_^−^ treatment [44,49].

Alternatively, higher NH_4_^+^ forms of nitrogen addition were positively correlated with S-ACPT and S-NiR and negatively correlated with soil pH, causing an antagonistic effect on soil as compared to NO_3_^−^. The variable effect of reduced and oxidized nitrogen addition in the same soil shows higher nutrient stability and improved soil health. For instance, high CO(NH_2_)_2_ significantly correlated with S-UE activity and stimulated total carbon content, promoting mineralization, but there was no significant effect of NH_4_^+^ and NO_3_^−^ fertilizers observed on S-UE activity in our results, which is similar to the findings of Li et al., who described non-significant effects of NH_4_^+^ and NO_3_^−^ on soil urease activity. Meanwhile, Weng et al. obtained the opposite results, in which applied NH_4_^+^ and NO_3_^−^ increased S-UE activity [50,51], and similarly study by Qu et al. also observed higher S-UE activity in *Larix olgensis* soil under high-level NO_3_^−^ addition. This discrepancy was possibly caused by differences in the available ammonia content, as our study shows higher nitrification, which resulted in decreased soil pH in the same way [52,53]. Therefore, the applied chemical nitrogen forms and levels improved *L. olgensis* soil nitrogen uptake efficiency and soil health, thereby stimulating beneficial microbial activities and soil functional properties.

### 4.2. Effect of Nitrogen Addition on AOA and AOB Abundances

As shown in this study’s results section, different forms and levels of nitrogen fertilization changed the abundance of AOB and AOA significantly. As expected, low NH_4_^+^ and low CO(NH_2_)_2_ as well as high NH_4_^+^ and high CO(NH_2_)_2_ impacted AOA and AOB as substrate sources and changed abundances. Previously, in different stable soil studies, the substrate level of AOA was already described, low NH_4_^+^ and low CO(NH_2_)_2_ in both acidic and alkaline soils increased AOA abundances [17,54]. Similarly, AOB increased abundances with high NH_4_^+^ application as a substrate [55]. But for the first time in oligotrophic forest soil, we obtained similar ammonia oxidizer abundance responses upon inorganic nitrogen application. In our study, AOA was higher under high CO(NH_2_)_2_, which may be due to mineralization or organic source availability [18].This reasoning is due to the significant increase in carbon (*p* ≤ 0.05) and non-significant correlation of high CO(NH_2_)_2_ treatment with total nitrogen in our results. Past studies have discussed how poor inorganic nitrogen conditions favor AOA as compared to AOB [56,57]; in the same way, in our results, AOA was negatively correlated with total nitrogen while AOB had a positive correlation, as described in Figure 3. Similarly, Figure 4 showed higher soil urease enzymatic activity (S-UE) correlation with AOA. This indicated influence of organic sources on AOA abundance. For instance, a study by Qin et al. also described that niche differentiation, between ammonia oxidizers caused by differences in substrate source affinity, in which AOA dominated AOB in high organic NH_3_^+^ availability [18].

AOB abundances under GL high-NH_4_^+^ treatment were significantly higher compared to all treatments (*p* ≤ 0.01), which correlates with previous studies’ results [13,58]. Zhaoming et al. obtained the same results with high-NH_4_^+^ application, in which overall AOB abundances increased while AOA was not affected by external N addition [16]. Similarly, Di et al. also described an increase in AOB as compared to AOA in high NH_4_^+^ concentration [59]. Although past studies reported that in low-pH soil, AOA activity increased as compared to AOB [60,61] but our study results oppose the hypothesis, that changes in soil pH contribute more to ammonia oxidizer distribution, which is a key point for oligotrophic forest soil nitrification pathways. At both the lowest soil pH and highest pH, AOA showed a significant lower abundance in GL and DX treatments. Correspondingly, a study in terrestrial grassland soil by Sterngren et al. demonstrated similar results to our study, in which AOA outnumbered AOB but AOB contributed relatively more in terms of ammonia oxidation in nitrogen-rich conditions [19]. In this experiment, the results for ammonia oxidizers were more influenced by nitrogen substrate source and applied concentrations, as can be seen in Figure 4; AOB positively correlated with NH_4_^+^ content. Generally, Acidic soil might be less favorable for AOA activity due to the protonation of ammonia. In the same way, numerous studies have found that high NH_4_^+^ has an increasing effect as a substrate on AOB [62,63] and a decreasing effect on AOA [64,65] regardless of soil type and vegetation [66,67].

### 4.3. Effect of Nitrogen Addition on nirK and nirS Abundances

Nitrite reduction is rate-limiting step for the denitrification process [68]. *nirK*-type and *nirS*-type denitrifiers, which are evolutionary different but have the same function, are involved in nitrite reduction [69]. A past study found that the *nirS* gene has more frequency of co-occurrence with *nosZ* and has the capability for complete denitrification as compared to *nirK* [70]. Thus, uncovering their adaptation ability in response to environmental change is ecologically important for N_2_O reduction. In previous studies, the effect of chemical nitrogen forms and levels on nitrite reductase abundances and the correlated impact on the potential rate of nitrogen conversion processes has been thoroughly elaborated in stable soil [24,71,72], but oligotrophic forest soil remains unexplored. We observed the direct and indirect effects of different synthetic nitrogen forms and levels on nitrite reductases in oligotrophic forest soil, and *nirK* gene abundances had shown greater increase upon low CO(NH_2_)_2_ treatment DN and high NO_3_^−^ GX treatment, while decreased under higher NH_4_^+^ GL treatment, correlating with the results of past studies [24,25]. This might be due to higher mineralization and higher urease enzyme activity, which provide more organic substrate for the *nirK* gene. In the results section (Figure 4), the *nirK* gene is correlated with soil urease activity (S-UE), which indicates a positive correlation with soil organic sources. Szukics et al. explained that the heterotrophic *nirK* gene is more adapted to organic conditions as compared to applied mineral nitrogen, ammonium and nitrate sources [73]. Comparatively, the *nirS* gene is more abundant in treatments where soil pH remained high, like DX, with lower NO_3_^−^ application, and less abundant in low pH at GL high NH_4_^+^ treatment. These different responses of nitrite reductases indicated different ranges of soil pH adaptability [43,74]. Our results correlated with the study by Herold et al., describing how nitrite reductases differentiated because of lower and higher soil pH [71]. Xu et al. also described how low soil pH decreases *nirS* gene abundance [75]; therefore, in our experimental soil, CO(NH_2_)_2_ and NH_4_^+^ with high and low levels had an inhibitory effect on *nirK* and *nirS*, thereby decreasing soil pH and consequently reducing denitrification [76]. Hallin et al. in their study also mentioned, variation of effects were caused due to different forms of nitrogen applied [32]. Furthermore, in the results section (Figure 3), soil nitrite reductase (S-NiR) activity negatively correlated with soil pH while *nirK* and *nirS* significantly positively correlated with pH [76,77].

Compared to ammonium-based nitrogen fertilizers, low NO_3_^−^ significantly increased *nirS* abundances (*p* ≤ 0.01) and was similarly statistically significant with water content (SWC), which indicated that *nirS* was more adapted to anaerobic conditions in this study as compared to *nirK*. Our results correlate with the studies by Wittorf et al. and Deng et al., describing how upon NO_3_^−^ addition, *nirS* gene abundances increase in anaerobic conditions [78,79]. Sarrenheimo et al. also examined *nirS* gene abundance under anaerobic conditions [80]. Similarly, Azziz et al. described lower activity of the *nirK* gene under anaerobic conditions [81]. As shown in Figure 4, the *nirS* gene’s strong correlation with soil pH, NO_3_^−^ content and SWC significantly increased under low-level NO_3_^−^ treatment, while *nirK* gene decreased with low NO_3_^−^ addition; this correlates with findings similar to the results of past studies [82,83]. We analyzed the relative abundances of genes by shot-gun metagenomic KEGG pathways, as shown in Figure 5, which strengthens our finding that the *nirK* gene is better adapted to organic conditions and less efficient to respond environmental changes as compared to *nirS*, which is higher in absolute abundances (qPCR) under chemical nitrogen addition. We concluded that although in the metabolic pathway, *nirK* was comparatively dominant in all nitrogen-applied treatments in *L. olgensis* soil due to its higher taxonomic diversity and adaptation of HGT (horizontal gene transfer) [43,84], inorganic N addition and anaerobic conditions shifted the nitrite reduction pathway to *nirS* gene [85,86]. Nadeau et al. found a similar association, in which *nirS* was positively correlated with areas of high soil moisture content, higher NO_3_^−^ concentration and stress conditions, while the *nirK* gene remained inefficient due to selective disadvantage [87]. Therefore, we consider that the detection limit of *nosZ* by qPCR quantification may be due to primer mismatch, as organisms possessing clade 1 *nosZ* were more likely to have the nitrite reductase gene than clade 11 *nosZ*, and the majority of *nosZ* reads from the shot-gun metagenome were likely clade 11, as it is much more phylogenetically diverse and tends to occur without nir [70]. Similarly, some studies also suggested that in anaerobic conditions, the direct competitors for NO_2_^−^ concentration are *NrfA* and *nirS* genes, leading to DNRA and denitrification pathways, respectively [88], in which *NrfA* is more abundant in high-carbon conditions [89] while the *nirS* gene is increased under higher NO_3_^−^ proportion [85].

Thus, our study demonstrated that fertilizer type and application level strongly influence the microbial pathways driving nitrogen cycling in acidic *L. olgensis* soils. Ecologically, such changes may affect soil nutrient availability, nitrogen retention and potential greenhouse gas emissions, with long-term consequences for forest productivity and sustainability. For oligotrophic forest management, our results suggest that carefully selecting the nitrogen fertilizer form and level is essential to maintain soil microbial balance, optimize tree growth and minimize nitrogen losses. These insights provide a scientific basis for developing more sustainable nitrogen fertilization strategies in nutrient-poor forest ecosystems.

## 5. Conclusions

This study focused on sustainable N transformation in oligotrophic forest soil, utilizing readily available mineral nitrogen sources. The application of varying levels of nitrogen forms had an overall positive effect on the soil nutrient status of *Larix olgensis*. The main factor affecting soil properties and enzymatic activities was high NO_3_^−^, which improved total nitrogen content and stabilized soil pH, thereby enhancing S-NAG and S-ACPT activities as compared to NH_4_^+^ and CO(NH_2_)_2_ fertilizers. From the perspective of fertilizer effect on microbial genes, the addition of NH_4_^+^ increased nitrification by stimulating NH_4_^+^ substrate concentration, shifting the ammonia oxidizer community to AOB, while despite the higher relative abundances of *nirK* and concurrent changes in soil moisture and soil pH, *nirS* gained the advantage of higher energetic costs, thus promoting complete denitrification. For future sustainable *Larix olgensis* growth, low-level ammonium fertilizer is suggested.

## Figures and Tables

**Table 1:** The impacts of nitrogen addition on the physico-chemical properties of soil.

Variable	pH	EC (ds m^−1^)	WC (%)	TC (g kg^−1^)	TN (g kg^−1^)	NH_4_^+^ (mg kg^−1^)	NO_3_^−^ (mg kg^−1^)
CK	5.613 ± 0.050 ^b^	44.300 ± 14.123 ^b^	9.467 ± 4.407 ^b^	37 ± 2 ^c^	3.1 ± 0.2 ^c^	1.230 ± 0.649 ^bc^	8.645 ± 3.522 ^cd^
GN	5.237 ± 0.051 ^c^	50.033 ± 15.130 ^b^	8.433 ± 0.493 ^ab^	39 ± 3 ^c^	3.7 ± 0.3 ^bc^	1.605 ± 0.366 ^bc^	6.075 ± 1.699 ^d^
DN	4.997 ± 0.129 ^c^	49.200 ± 9.350 ^b^	10.967 ± 3.465 ^b^	41 ± 1 ^bc^	4.8 ± 1 ^ab^	2.380 ± 1.036 ^a^	9.112 ± 2.648 ^bc^
GL	4.563 ± 0.170 ^d^	42.367 ± 4.842 ^a^	10.500 ± 0.794 ^ab^	45 ± 3 ^a^	5.2 ± 2 ^ab^	1.549 ± 0.527 ^b^	7.618 ± 3.609 ^b^
DL	5.107 ± 0.055 ^c^	45.500 ± 5.556 ^ab^	12.333 ± 4.989 ^b^	40 ± 5 ^a^	5.0 ± 0.1 ^a^	1.084 ± 0.443 ^bc^	7.621 ± 1.130 ^bcd^
GX	5.837 ± 0.093 ^ab^	89.833 ± 18.095 ^b^	8.533 ± 2.301 ^b^	49 ± 1 ^ab^	5.6 ± 5 ^a^	−0.020 ± 0.133 ^d^	16.381 ± 4.323 ^a^
DX	5.990 ± 0.017 ^a^	53.300 ± 5.129 ^b^	10.433 ± 5.105 ^a^	29 ± 4 ^bc^	4.8 ± 0.2 ^ab^	1.237 ± 0.145 ^cd^	7.553 ± 0.730 ^bcd^

Note: GN: High-level urea treatment. DN: Low-level urea treatment. GL: High-level ammonium chloride treatment. DL: Low-level ammonium chloride treatment. GX: High-level sodium nitrate treatment. DX: Low-level sodium nitrate treatment. CK: Control treatment. EC: electrical conductivity. WC: water content. TC: total carbon. TN: total nitrogen. Different letters within a coloumn (or row) indicate significant differences while identical letters indicate no significant differences.

## Data Availability

The raw data supporting the conclusions of this article will be made available by the corresponding author upon request.
